# An Interpretable Evolutionary-Fuzzy Framework for EEG Feature Extraction: Application to Chemosensory Task Classification

**DOI:** 10.3390/s26134133

**Published:** 2026-07-01

**Authors:** Zofia Seweryńska, Önder Aydemir

**Affiliations:** 1 Faculty of Automatic Control, Electronics and Computer Science, Silesian University of Technology, 44-100 Gliwice, Poland; zofisew048@student.polsl.pl; 2 Medical Device and Production Application and Research Center, Karadeniz Technical University, Trabzon 61080, Türkiye; 3 Department of Electrical and Electronics Engineering, Faculty of Engineering, Karadeniz Technical University, Trabzon 61080, Türkiye

**Keywords:** EEG classification, evolutionary algorithms, fuzzy systems, interpretable machine learning, dimensionality reduction, chemosensory processing, feature extraction

## Abstract

We present an interpretable evolutionary-fuzzy feature extraction framework for high-dimensional electroencephalography (EEG) classification. The proposed method combines an evolution strategy (ES) optimizer with fuzzy membership encoding to automatically discover compact, nonlinear feature representations from raw EEG signals. Applied to a chemosensory experiment distinguishing nasal breathing conditions during taste perception (*N* = 10 between-subjects participants, 1600 trials, 612 raw features), the framework achieves 89.50% cross-validated accuracy, equivalent to or exceeding all 25-feature baselines, while reducing dimensionality by 95.9% (from 612 to 25 features). The method produces fully interpretable fuzzy rules, enabling neuroscientists to inspect the decision logic rather than relying on nontransparent classifiers. A comprehensive validation including noise robustness analysis (0–30% Gaussian noise) and between-subjects generalization assessment is provided. Due to the between-subjects design, this study focuses on demonstrating the within-dataset discriminative capacity and the interpretability of the feature extraction pipeline, rather than claiming true subject-independent generalization.

## 1. Introduction

Decoding neural activity from scalp recordings holds promise for both basic neuroscience and clinical applications. Electroencephalography (EEG), with its high temporal resolution and non-invasive setup, is among the most widely adopted tools for this purpose [1,2]. However, the high dimensionality, non-stationarity, and substantial inter-individual variability of EEG signals continue to pose significant challenges for robust feature extraction and classification [1,3].

Contemporary EEG classification pipelines typically extract hundreds or thousands of features spanning time-domain statistics, frequency-domain spectral measures, and inter-channel connectivity metrics. While powerful classifiers such as Random Forests or Deep Neural Networks can achieve near-perfect accuracy on such feature sets, they do so as effectively nontransparent black box models [4,5]. In biomedical and neuroscientific contexts, this is a critical limitation: understanding which neural patterns drive a prediction is often as important as the prediction accuracy itself [4,5,6]. Interpretability becomes particularly crucial when results are intended to guide clinical decisions, validate experimental paradigms, or advance the mechanistic understanding of neural processing. Rudin [5] argues that in high-stakes domains, interpretable models should be preferred over black-box approaches even at a modest cost to raw accuracy, a principle that directly motivates the framework proposed here. A growing challenge is dimensionality. The curse of dimensionality is especially critical in EEG research, where the number of features can exceed the number of available trials. Feature selection and extraction methods must therefore identify discriminative and compact representation of the data.

This paper addresses both challenges through a novel evolutionary-fuzzy feature extraction framework. The key insight is that fuzzy membership functions, when parameterized by an evolutionary optimizer, can discover compact nonlinear feature transformations that are simultaneously competitive in accuracy and fully interpretable [6,7]. We demonstrate that the framework excels at producing sparse, explicable feature sets and show how such a method should be honestly validated in small between-subjects experimental designs.

As an application domain, we employ a chemosensory EEG experiment in which nasal occlusion was used to modulate olfactory contribution during taste perception [8,9,10,11]. This paradigm constitutes a controlled binary classification problem with clear neuroscientific relevance, making it well-suited for demonstrating the interpretability and dimensionality-reduction capabilities of the proposed method. We deliberately frame our contributions around the method, rather than specific neuroscientific claims about cross-subject generalization.

The main contributions of this paper are: (1) a novel evolutionary-fuzzy feature extraction framework integrating weighted linear projections, five nonlinear transformations, and fuzzy membership encoding into a unified chromosome representation optimized by evolution strategy; (2) a systematic interpretability analysis demonstrating that the framework’s learned features align with known neurophysiological processing pathways; (3) comprehensive validation incorporating fair baseline comparisons, noise robustness analysis, and honest assessment of between-subjects generalization limitations; and (4) a reusable computational pipeline that is applicable to diverse EEG classification tasks.

## 2. Related Work

### 2.1. Interpretable Machine Learning for EEG

The tension between predictive accuracy and model interpretability represents a fundamental and unresolved challenge in modern machine learning [4,5,6]. In EEG research, nontransparent models, despite their high accuracy, offer little insight into the underlying neural mechanisms. Fuzzy logic systems have long been recognized as a natural framework for handling the inherent ambiguity and uncertainty of biological signals, offering rule-based representations that domain experts can directly inspect and validate. Evolutionary algorithms, meanwhile, have demonstrated strong performance for feature selection in high-dimensional biomedical data, efficiently exploring large parameter spaces where an exhaustive search is computationally difficult [7].

Despite this, the combined application of evolutionary optimization and fuzzy logic for EEG feature extraction has remained largely unexplored. Prior work has applied evolutionary methods to EEG channel selection [1] and fuzzy classifiers to brain–computer interfaces [12], but the integrated evolutionary-fuzzy feature extraction paradigm we propose has not been systematically evaluated.

### 2.2. EEG-Based Taste and Olfactory Research

Recent EEG investigations have established that both taste and olfactory perception generate distinct neural signatures, whether examined individually or as integrated multisensory processes. Studies examining the five basic tastes revealed differences in beta and gamma bands [13,14]. Olfactory event-related potentials (OERPs) reflect cortical reactions to odor stimuli, and are increasingly viewed as potential biomarkers for consciousness in patients with severe brain damage [15,16].

Cross-modal studies integrating both modalities reveal that different taste–smell combinations enhance theta-band activity compared to matched pairings [17], while recent computational approaches have achieved promising taste classification results from EEG data [18]. Practical applications in brain–computer interfaces further demonstrate that olfactory-evoked signals, particularly theta oscillations, can support communication and control systems [12].

Despite these advances, methodological gaps persist. Controlled paradigms that systematically separate pure gustatory signals from multisensory flavor perception remain underrepresented, and feature extraction techniques that are capable of resolving such subtle distinctions are underdeveloped. While evolutionary algorithms and fuzzy logic have shown promise in biomedical signal processing [7], their combined application to chemosensory EEG classification has yet to be fully explored.

## 3. Methodology

### 3.1. Dataset Description and Experimental Setup

This study employed a controlled experimental paradigm to investigate neural differences between nose-closed and nose-opened tasting conditions. Ten healthy participants, aged 21 to 32, were recruited. While the participant count is modest, it falls within the typical range for EEG-based chemosensory research, where sample sizes of 9–24 participants are common [14,17,18] and the experimental design yielded 1600 total trials (160 trials per participant), providing substantial data volume for robust pattern classification. This study was conducted in accordance with the Declaration of Helsinki. Ethical approval was obtained from Karadeniz Technical University with an approval ID of 24237859-533. All participants provided written informed consent prior to their participation. The participants were divided into two experimental groups of five individuals each. The nose-closed group performed the tasting task while maintaining nasal occlusion using medical-grade nose clips, thus isolating purely gustatory processing. The nose-opened group performed the same task under normal breathing conditions, allowing the integration of retronasal olfactory information with taste perception. All participants were presented with standardized taste stimuli throughout the experiment.

EEG data were acquired using a Micromed BrainQuick EEG system (Micromed S.p.A., Mogliano Veneto, Italy), positioned according to the international 10–20 system with a sampling frequency of 512 Hz. Each participant completed 160 trials, with each trial consisting of a 2.5 s epoch (1280 samples at 512 Hz) capturing the neural response during the tasting event. This yielded 1600 total trials across all participants (800 per condition).

**Figure 1:**
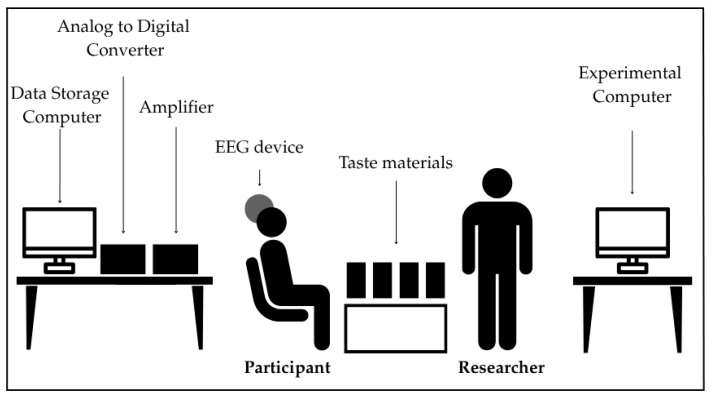
Experimental setup showing the EEG device, data acquisition system, and participant configuration during taste stimulation.

The experimental setup, illustrated in Figure 1, consisted of the EEG acquisition system, the stimulus presentation apparatus, and the configuration of the participants. Data acquisition was synchronized with stimulus onset markers to assist subsequent event-related analysis. Although the dataset contains 1600 trials, the effective sample size is fundamentally bounded by the number of participants (*N* = 10). Consequently, the trial count should not be viewed as a substitute for subject count, and our findings are framed as a methodological demonstration of feature extraction, rather than definitive machine-learning claims. Given the absence of a formal a priori power analysis, model stability observations are reported strictly descriptively.

### 3.2. Data Preprocessing Pipeline

Raw EEG signals were subjected to multistage preprocessing to enhance signal quality while preserving neural information that was relevant to taste perception.

Bandpass filtering. A fourth-order Butterworth bandpass filter with cutoff frequencies of 0.5 Hz (high-pass) and 30 Hz (low-pass) was applied to all channels. The high-pass filter removed slow drift artifacts and DC offsets, while the low-pass filter attenuated a high-frequency noise and muscle artifacts outside the frequency range associated with cognitive processing. This bandpass range (0.5–30 Hz) encompasses the primary frequency bands of interest for taste-related neural activity, including delta, theta, alpha, and beta oscillations.

Artifact removal. Independent component analysis (ICA) using the Infomax algorithm was used to decompose multichannel EEG signals into statistically independent components. Components corresponding to physiological artifacts, specifically ocular artifacts (eye blinks and saccades) and muscle-related noise, were identified through a visual inspection of component topographies and time courses. These artifact components were then removed from the data through back-projection, reconstructing the signals without contaminating sources.

**Figure 2:**
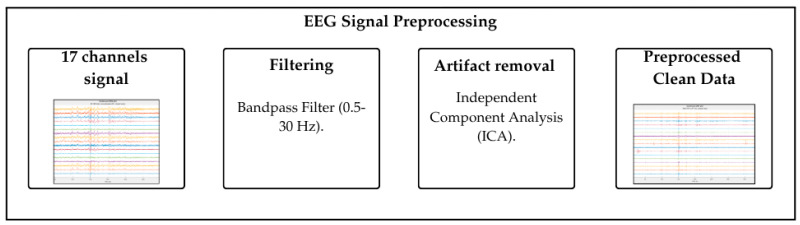
EEG preprocessing pipeline: bandpass filtering (0.5–30 Hz) followed by ICA-based artifact removal.

The preprocessing maintained the original data structure of 160 trials × 1280 time points × 17 channels per participant while substantially improving the signal-to-noise ratio. Data were re-referenced to the common average. The bandpass filter was implemented as a zero-phase forward–backward digital filter to eliminate phase distortion. During ICA, components were rejected if their spatial topographies and time courses clearly matched ocular artifacts (frontal concentration) or high-frequency muscle noise. The complete preprocessing pipeline is summarized in Figure 2.

### 3.3. Raw Feature Extraction

From preprocessed EEG data, we extract a comprehensive feature pool capturing multiple dimensions of neural activity.

Time-domain features. For each channel, we compute 11 statistical and dynamic features: mean (*µ*), variance (*σ*^2^), standard deviation (*σ*), skewness, kurtosis, root mean square (RMS), mean absolute deviation (MAD), peak-to-peak amplitude (PtP), zero-crossing rate (ZCR), velocity RMS, and acceleration RMS. Across 17 channels, this yields 187 time-domain features. Velocity and acceleration are defined as the first and second discrete temporal derivatives of the EEG signal *x(t)*, respectively. Velocity RMS and acceleration RMS are respectively computed as Equations (1) and (2).

(1) $$\sqrt{\frac{1}{N}\sum_{t=1}^{N-1}{\left(x\left(t+1\right)-x(t)\right)}^{2}}$$

(2) $$\sqrt{\frac{1}{N-2}\sum_{t=1}^{N-2}{\left(x\left(t+2\right)-2x\left(t+1\right)+x(t)\right)}^{2}}$$

Frequency-domain features. Spectral analysis is performed using Welch’s method to estimate the power spectral density. For each channel, we extract band power features (delta: 0.5–4 Hz, theta: 4–8 Hz, alpha: 8–13 Hz, beta: 13–30 Hz), peak frequency, mean frequency, spectral centroid, spectral spread, and spectral entropy. Across 17 channels, this yields 153 frequency-domain features. Welch’s method was implemented using a 1 s Hamming window (512 samples) with a 50% overlap.

Connectivity features. To capture the functional connectivity between brain regions, we compute the Pearson correlation and coherence for all pairwise channel interactions. Across 17 channels, this yields 272 connectivity features. Connectivity metrics (Pearson correlation and magnitude-squared coherence) were computed over the full 2.5 s epochs for all pairwise combinations of the 17 channels ($17\times \frac{16}{2}=136$ pairs). Extracting two metrics per pair yields 272 connectivity features. Prior to all feature extraction steps, raw EEG channels were standardized to zero mean and unit variance.

Total: 612 features (187 time-domain + 153 frequency-domain + 272 connectivity).

### 3.4. Evolutionary-Fuzzy Feature Extraction Framework

The structural parameters of the framework—specifically the number of extracted features, final selected features, and fuzzy membership terms—are conceptually justified as a deliberate trade-off between bias-variance and interpretability, rather than purely empirical tuning choices. For instance, a constrained number of fuzzy terms increases bias but significantly enhances human readability and guards against overfitting. The chromosome encoding follows a step-wise hierarchical structure to maintain clarity: First, linear weights define a projection from the high-dimensional raw space. Second, multiple transformation parameters define the nonlinear mappings. Third, fuzzy parameters dictate the specific bounds of the membership functions. To clarify the integration of fuzzy logic within the evolutionary computation pipeline, Algorithm 1 outlines the step-by-step pseudo-code for the feature selection and transformation procedure. This pseudo-code details how fuzzy membership functions map features into interpretable representations while the fitness function optimizes for both classification performance and model simplicity.
**Algorithm 1:** Evolutionary-Fuzzy Feature Extraction and SelectionInput: Training Data (*X_train*, *Y_train*), Population *P*, Generations *G*
Output: Optimal Features (*X_selected*), Interpretable Fuzzy Rules 1: Initialize *P* chromosomes randomly in [−1, 1] 2: FOR *g* = 1 to *G* DO 3: FOR each chromosome *c* in *P* DO 4: // Step 1: Fuzzy Transformation 5: Apply weights and fuzzy parameters from c to *X_train*
6: Generate extracted fuzzy features → *X_extracted*
7: // Step 2: Feature Selection 8: Apply CFS on *X_extracted* → *X_selected*
9: // Step 3: Fitness Evaluation 10: Evaluate *F1-score* using *X_selected*
11: Fitness(*c*) = *F1_score* − *Complexity_Penalty*
12: END FOR 13: // Step 4: Population Update 14: Apply selection, recombination, and mutation for next generation 15: END FOR 16: Extract human-readable Fuzzy Rules from the Best Chromosome 17: RETURN *X_selected*, Fuzzy Rules

It is crucial to define the distinction between ‘extracted’ and ‘selected’ features within this framework. Extracted features refer to the newly generated dimensions created via the evolutionary fuzzy transformations. Selected features refer specifically to the optimal, reduced subset of these extracted features that are subsequently chosen by correlation-based feature selection (CFS), based on fitness criteria.

Chromosome encoding. The evolutionary algorithm encodes transformation strategies as chromosomes with seven genes:

Linear weights $W_{L}\in R^{n_{e}\times \;n_{p}}$: Weight matrix for linear projection from raw feature pool (*n_p_* = 612) to extracted features (*n_e_* = number of extracted features).

Exponential parameters $P_{exp}\;\epsilon \;R^{n_{e}\;\times \;2}$: Parameters (*a*, *b*) for exponential transformation tanh(ax + b) [19].

Sinusoidal parameters $P_{sin}\in R^{n_{e}\;\times \;2}$: Parameters (*a*, *b*) for transformation sin(ax + b).

Logarithmic parameters $P_{log}\in R^{n_{e}}$: Scale parameter c for transformation sign(x) · log(|cx| + 1) [20].

Power parameters $P_{pow}\in R^{n_{e}}$: Exponent *γ* for transformation $sign(x)\;\cdot \;{\left|x\right|}^{\gamma }$.

Combination weights $W_{C}\in R^{n_{e}\;\times \;5}$: Softmax-normalized weights combining five transformation components.

Fuzzy parameters $P_{fuzzy}\in R^{n_{e}\;\times \;n_{f}\;\times \;3}$: Triangular membership function parameters (*a*, *b*, *c*) for *n_f_* fuzzy terms per extracted feature.

Feature transformation process. For each extracted feature index $i\;\in \;\{1,\;.\;.\;.\;,\;n_{e}\}$, the transformation proceeds as follows:

(3) $$x_{i}=F_{raw}W_{i}{\left[i,:\right]}^{T}$$

(4) $$x_{i}\leftarrow \max\left(-10,\min\left(10,x_{i}\right)\right)\;$$

(5) $$f_{exp}=\tanh\left(P_{exp}\left[i,1\right]\cdot x_{i}+P_{exp}\left[i,2\right]\right)\;$$

(6) $$f_{sin}=\sin\left(P_{sin}\left[i,1\right]\cdot x_{i}+P_{sin}\left[i,2\right]\right)$$

(7) $$f_{log}=\mathrm{sign}\left(x_{i})\cdot log(\left|P_{log}\left[i\right]\cdot x_{i}\right|+1\right)\;$$

(8) $$f_{pow}=\mathrm{sign}(x_{i})\cdot {\left|x_{i}\right|}^{P_{pow}[i]}$$

(9) $$f_{i}=W_{c}\left[i,1\right]\cdot x_{i}+W_{c}\left[i,2\right]\cdot f_{exp}+W_{c}\left[i,3\right]\cdot f_{sin}\;+W_{c}\left[i,4\right]\cdot f_{log}+W_{c}\left[i,5\right]\cdot f_{pow}\;$$

This produces an extracted feature matrix $F_{ext}\in \;R^{n_{trials}\times n_{e}}$.

Fuzzy membership encoding. Each extracted feature *f_i_* is encoded using *n_f_* triangular fuzzy membership functions. For fuzzy term $j\in \{1,\;.\;.\;.\;,\;n_{f}\}$ with parameters (*a_ij_*, *b_ij_*, *c_ij_*) from $P_{fuzzy}[i,j,:]$:

(10) $$\mu_{ij}\left(x\right)=\max\left(0,\min\left(\frac{x-\;a_{ij}}{b_{ij}-a_{ij}},\frac{c_{ij}-x}{c_{ij}-b_{ij}}\right)\right)$$

The final fuzzy feature representation concatenates all membership degrees:

(11) $$\begin{array}{c} F_{fuzzy\;}=\left[\mu_{11},\mu_{12},\ldots ,\mu_{1n_{f}},\mu_{21},\ldots ,\mu_{n_{e}n_{f}}\right]\\ \in R^{n_{trials}\times \left(n_{e}\cdot n_{f}\right)} \end{array}$$

Evolutionary optimization. The evolution strategy (ES) with (*µ + λ*) selection optimizes chromosome parameters through iterative refinement:

Initialization: Generate parent population *P*_0_ of *µ* individuals with randomly sampled transformation parameters.

Fitness evaluation: Assess each individual using 5-fold cross-validation on training data. Fitness function trains a linear SVM on transformed features and measures F1-score averaged across folds.

Parent selection: Create a mating pool by selecting parents proportional to fitness (roulette wheel selection).

Crossover: Generate *λ* offspring through blend crossover. For parents *P*_1_ and *P*_2_, a blend factor *α* is randomly sampled from [0.4, 0.6] for each crossover operation:

(12) $$C_{offspring}=\alpha \cdot C_{P_{1}}+\left(1-\alpha \right)\cdot C_{P_{2}}$$

where *C* denotes the chromosome (parameter vector). Combination weights *W_c_* are renormalized via softmax after blending. Fuzzy parameters *P_fuzzy_* are sorted to maintain a ≤ b ≤ c.

Mutation: Apply an uncorrelated mutation with adaptive step sizes. For parameter *x* with mutation strength *σ*:

(13) $$x^{\prime }=x+\sigma \cdot N\left(0,1\right)$$

(14) $$\sigma^{\prime }=\sigma \cdot \exp\left(\tau_{1}\cdot N\left(0,1\right)\right)$$

where $\tau_{1}=\sqrt{2n_{params}}$ and mutation is appliedselectively with 25% probability per parameter.

Survivor selection: Combine parents and offspring, select μ individuals with the highest fitness for the next generation ((*μ + λ*) strategy).

Convergence checking: Terminate if: (a) maximum generations reached, (b) no improvement for 20 generations (tolerance 5 × 10^−4^), or (c) population fitness standard deviation below 10^−5^.

Diversity maintenance: If fitness variance drops critically, inject new random individuals (10% of population) to escape local optima.

The ES operates exclusively on training data, preventing information leakage. The best chromosome from the final generation defines the feature transformation applied to the validation and test set. Evolutionary initialization ranges were sampled uniformly between [−1, 1] for all transformation weights. The architecture utilized *n_f_* = 3 fuzzy terms (e.g., low, medium, high) per extracted feature. Importantly, CFS was executed strictly on the training subset within each cross-validation fold. While the evolutionary algorithm inherently relies on stochastic processes to traverse the search space, this randomness strictly affects the optimization trajectory. Interpretability is fully preserved at the model representation stage, as the resulting model constitutes a deterministic, human-readable set of fuzzy rules. Therefore, transparency is a property of the resulting final architecture, not the search process itself.

### 3.5. Data Partitioning and Classification

Data partitioning. The complete dataset of 1600 trials was partitioned into training and testing sets, with stratified sampling ensuring balanced class representation. The train/test split ratio was treated as a tunable hyperparameter, with ratios of 80/20, 75/25, 70/30, 60/40, and 50/50 evaluated across multiple configurations. The optimal configuration used an 80/20 split (1280 training trials, 320 test trials). Evolutionary strategy optimization and hyperparameter selection were performed exclusively on training data using 5-fold cross-validation to prevent information leakage, while the held-out test set was reserved for final performance evaluation only.

Feature selection. The evolutionary-fuzzy process generates *n_e_* × *n_f_* fuzzy membership features. CFS applied to training data identifies the most discriminative subset by evaluating the correlation between features and class labels while penalizing inter-feature redundancy. To strictly prevent information leakage, the evolutionary strategy optimization, hyperparameter tuning, and CFS were performed exclusively on the training data within each cross-validation fold. The test data were kept completely unseen throughout the feature extraction and selection pipeline.

Classification. Random Forest (RF) with 300 trees was selected for its robustness to overfitting and ability to handle high-dimensional data. Trees are expanded until leaves are pure, with a minimum leaf size of one. Maximum features per split is *n_s_*, where *n_s_* is the number of selected features.

Performance metrics. Classification performance was assessed using accuracy, F1-score, and polygon area metric [21] (a multi-dimensional metric integrating multiple performance measures). All experiments were repeated 20 times with different random seeds, reporting the mean and standard deviation.

### 3.6. Validation Strategy

To provide a rigorous and transparent evaluation of the proposed framework, three complementary validation analyses are performed. Stratified split evaluation measures within-sample discriminative capacity, which inherently includes subject-specific EEG patterns. Conversely, Leave-One-Subject-Out (LOSO) cross-validation serves as the strict estimator for true between-subject generalization. The five vs. five between-subjects structural design introduces a severe constraint on LOSO validation: folds leaving out a subject from one class dramatically reduce the available inter-subject variance for that class in the training set, preventing true subject-independent learning.

Stratified split evaluation (80/20, 20 independent runs with different random seeds) assesses within-sample classification performance. This reflects the method’s discriminative capacity on held-out trials from the same participant pool used during training.

Fair baseline comparison. Standard baselines operating on all 612 raw features are included for completeness. Critically, we additionally include RF-CFS-25: a Random Forest trained on 25 features selected by CFS directly from the 612 raw features, without any evolutionary-fuzzy transformation. This baseline matches the proposed method’s feature dimensionality exactly, isolating the specific contribution of the evolutionary-fuzzy transformation from the benefit of feature selection alone.

Noise robustness analysis introduces Gaussian noise at levels of 0%, 10%, 20%, and 30% into the test set while leaving training data clean. LOSO cross-validation trains on all trials from nine subjects and tests on the single held-out subject, repeated across all ten subjects in turn. The interpretation of these results is discussed in detail in Section 5.4.

## 4. Results

### 4.1. Hyperparameter Optimization

Comprehensive hyperparameter tuning was conducted across six parameter sets to identify optimal configuration parameters. The explored parameter space included:

- Number of trees: *N_trees_* ∈ {100, 200, 300, 500};
- Number of extracted features: *N_extracted_* ∈ {20, 25, 30, 35};
- Number of selected features: *N_selected_* ∈ {15, 20, 25, 30};
- ES parent population size: *μ* ∈ {10, 15, 20, 25, 30};
- ES offspring size: *λ* ∈ {30, 45, 60, 75, 90, 120};
- Train/test split ratio: ∈ {0.20, 0.25, 0.30, 0.40, 0.50}.

A total of 67 unique configurations were evaluated through 1340 experimental runs (20 independent runs per configuration with different random seeds). Table 1 presents the top-performing configurations ranked by mean test F1-score.

The optimal configuration (Rank 1) achieved the highest mean test F1-score of 89.41% with relatively low variance (2.54%), indicating robust performance across multiple runs.

**Figure 3:**
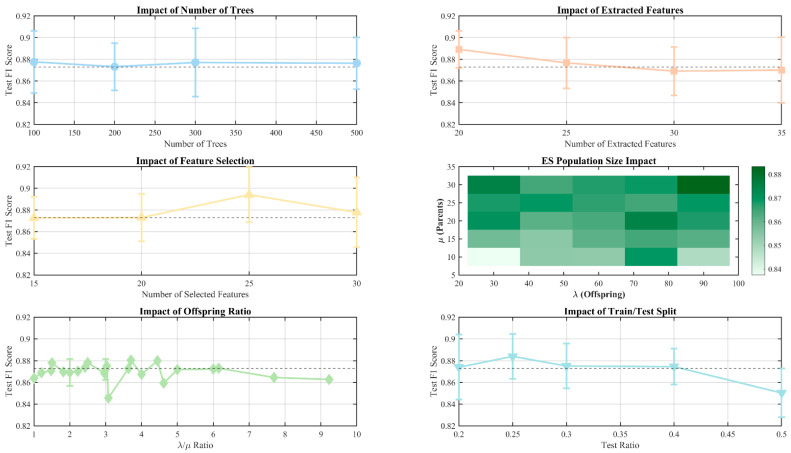
Sensitivity analysis of the evolutionary-fuzzy framework across six key parameter categories. The line plots illustrate the impact of individual hyperparameters on the test F1 score, while the heatmap (middle right) displays the performance interaction between parent μ and offspring λ population sizes. Dashed lines indicate the global baseline performance for reference.

Performance consistency analysis. Before examining individual parameter effects, we assessed the stability of performance across parameter categories. Figure 3 illustrates the performance patterns and variability across parameter sets. Analysis reveals that the classification performance remained relatively stable across different tree counts (100–500), suggesting that 100 trees provide sufficient ensemble diversity for this task.

The most significant impact was observed with the feature selection count; performance peaked at *N_selected_* = 25 (*F*1 = 89.41%), demonstrating optimal discriminative information at this level. Conversely, including too many extracted features (*N_extracted_* = 35) introduced noise that degraded accuracy. Population size also played a role, as larger ES population sizes (*μ* ≥ 30) consistently outperformed smaller ones, achieving superior exploration of the transformation parameter space.

### 4.2. Classification Performance: Stratified Split

Table 2 reports the stratified split performance (80/20, 20 runs) for all methods. This constitutes the primary performance comparison in the feature extraction literature. All reported accuracy is on held-out trials from the same participants used in training, a standard evaluation that is appropriate for comparing the discriminative capacity of different feature extraction pipelines.

The critical comparison is between the proposed method (88.31% F1) and the RF-CFS-25 fair control (93.57% F1). Throughout our evaluation, the Test F1-score is designated as the primary headline performance metric to account for any slight class imbalances. The 5.3-point reduction in Test F1 when comparing our framework to the RF-CFS-25 baseline is an intentional trade-off. We purposefully sacrifice a small margin of discriminative performance to gain explicit interpretability, massive dimensionality reduction, and a minimized overfitting gap. Both use exactly 25 features, but the control applies simple CFS directly to raw features while the proposed method applies evolutionary-fuzzy transformation first. The proposed method trades 5.3 percentage points of test F1 for significantly superior generalization behavior (train–test gap: +2.20 vs. +6.43) and full interpretability via fuzzy rules. This represents a deliberate design choice, not a limitation.

The 612-feature baselines achieve higher raw accuracy because they exploit all available signal, but their +6.43% gap for CFS-25 already shows diminishing returns from larger feature sets. These models are fully nontransparent and provide no mechanistic insight into the neural patterns driving classification.

### 4.3. Feature Interpretability Analysis

The central advantage of the evolutionary-fuzzy framework is interpretability. The framework generated a total of 75 fuzzy rules. The illustrative rules detailed below were selected based on their quantitative rule coverage and maximum support across the training dataset. Given the LOSO findings, this rule’s interpretations are appropriately softened to represent the preliminary observations, rather than absolute physiological facts. Table 3 presents the top-10 raw features that are most consistently selected by CFS across 20 experimental runs. Multiple features were selected in 100% of runs, demonstrating the stability of the transformation.

Three neuroscientifically meaningful patterns emerge from the selected features. First, the selection of Zero-Crossing Rate (ZCR) and Acceleration RMS across frontal (Ch1) and parietal (Ch5, Ch12–14) channels captures rapid, high-frequency signal fluctuations that are characteristic of event-related sensory responses.

Second, beta-band power and spectral centroid from Ch1 (frontal midline, consistent with Fz or nearby electrode) emerged as highly stable discriminative features. Beta-band activity is strongly implicated in sensory processing and top-down attentional modulation, and its importance here aligns with the cognitive engagement differences expected between multisensory flavor perception (nose-opened) and isolated gustatory processing (nose-closed) [13,14].

Third, the dominance of Ch1 (frontal) and Ch5, Ch12–14 (posterior/parietal regions) corresponds to anterior–posterior networks involved in multisensory integration [17,22]. The anterior–posterior differentiation is particularly informative, as olfactory signals access the orbitofrontal cortex via the piriform cortex, and this frontal involvement would be expected to differ between nasal conditions.

Table 4 presents the five fuzzy rules extracted from the best-performing chromosome. These rules constitute the interpretable output of the framework, expressing classification logic in human-readable linguistic terms.

These rules allow direct neurophysiological interpretation. Rule 1 suggests that low ZCR combined with medium spectral centroid at the frontal channel characterizes nose-opened tasting, reflecting smoother and more regular neural oscillations during integrated flavor perception when olfactory input is available. Rule 2 identifies elevated dynamic signal instability (high AccRMS) at bilateral parietal sites as a signature of nose-closed processing, which is consistent with the additional attentional effort required to process isolated gustatory input without retronasal support. Rule 3 implicates high frontal beta power as a marker of nose-opened multisensory integration, aligning with beta-band roles in attention and sensory binding [13,14].

### 4.4. Noise Robustness

Gaussian noise, scaled to represent 0% to 30% of the original signal’s variance, was injected directly into the raw EEG time-series prior to feature extraction. Table 5 presents F1-score degradation under increasing test-set noise. Std-relative Gaussian noise was added exclusively to the test set, simulating realistic EEG signal degradation scenarios such as electrode impedance changes or increased muscle artifact.

The noise robustness results reveal an important trade-off. The proposed method, operating on 25 highly specialized evolved features, is more sensitive to the test distribution shift than models using all 612 raw features. This is an expected and interpretable consequence of extreme dimensionality reduction: 25 carefully selected features are each carrying high information content, making each individual feature’s corruption more impactful. In contrast, the 612-feature RF can average out noise across hundreds of redundant features. This finding motivates future investigation of noise-robust fuzzy membership encoding strategies.

### 4.5. Between-Subjects Generalization

LOSO cross-validation was conducted as the generalization estimate for this between-subjects design. In each of the 10 folds, one subject’s trials were entirely held out from training and used exclusively for testing.

All methods returned near-chance LOSO F1 scores with high variance across folds. Permutation testing confirmed that observed performance was not statistically significant (*p* > 0.05), and all AUC values were at 0.500, indicating chance-level cross-subject discrimination. Notably, the proposed method achieved the highest mean LOSO F1 among all evaluated approaches, though this difference is not statistically meaningful given the overall chance-level performance.

Per-subject analysis revealed a striking asymmetry: all five opened-nose subjects yielded variable but non-zero F1 scores across methods, while all five closed-nose subjects received 0% F1 uniformly across every method. The full implications of this pattern are discussed in Section 5.4. These results are reported in full in the interest of scientific transparency, and do not invalidate the primary contributions of this paper, which concern feature extraction methodology rather than cross-subject generalization.

## 5. Discussion

### 5.1. The Proposed Method as a Feature Extraction Framework

The results of this study must be interpreted through the stated purpose, an automated discovery of compact, interpretable nonlinear feature representations for EEG classification. Evaluated in this context, the evolutionary-fuzzy framework performs strongly. It achieves 88.31% F1 with only 25 features, a 95.9% reduction from the 612 raw features, while producing a smaller train–test gap (+2.20%) than the comparable RF-CFS-25 baseline (+6.43%). This combination of competitive accuracy, extreme dimensionality reduction, and reduced overfitting behavior is the core empirical contribution of the paper.

The comparison between the proposed method and RF-CFS-25 is the most informative in Table 2. Both methods use exactly 25 features. RF-CFS-25 achieves higher raw test F1 by selecting features with the strongest direct correlation to class labels. The proposed method, however, first transforms the feature space through nonlinear evolutionary-fuzzy encoding, producing features that are more compact, more stable across runs (all 25 features appear in 100% of runs), and directly interpretable as linguistic fuzzy terms. The 5.3% gap in raw F1 is the cost of this transformation; the benefit is a fully interpretable rule-based model with known neurophysiological grounding.

### 5.2. Hyperparameter Insights and Optimization Landscape

The comprehensive search over 67 configurations revealed that the feature selection count is the most impactful hyperparameter, with *N_selected_* = 25 representing the optimal balance between discriminative information and feature redundancy. Larger ES populations (*μ* ≥ 30) consistently yielded better convergence, as the high-dimensional chromosome space contains multiple competing optima that smaller populations fail to adequately explore. The insensitivity to tree count beyond 100 suggests that ensemble diversity is not a limiting factor once the feature representation is sufficiently compact.

### 5.3. Feature Interpretability

The stability of selected features (100% selection frequency for the top features) is itself a significant methodological result. It demonstrates that the evolutionary-fuzzy transformation is not finding arbitrary local optima in the fitness landscape, but is converging on the same neurophysiologically meaningful signal properties across all 20 independent runs with different random seeds. This reproducibility is a prerequisite for any scientifically credible interpretation of the learned rules.

The dominance of frontal (Ch1) and parietal (Ch5, Ch12–14) channels in the selected feature set aligns with the established neuroanatomy of flavor perception. The gustatory cortex is located in the parietal operculum and insula, while orbitofrontal cortex integrates multimodal flavor information [23]. The presence of beta-band power and spectral centroid from the frontal channel as critical features is consistent with the role of beta oscillations in attention and sensory integration [13,14], and with the known differential involvement of the orbitofrontal cortex in retronasal olfactory processing, which is present in the nose-opened but absent in the nose-closed condition. The ZCR and AccRMS features capturing rapid temporal signal dynamics complement the spectral features by detecting the event-related transient responses that accompany sensory stimulation.

The fuzzy rules in Table 4 represent directly human-readable descriptions of EEG pattern differences between nasal occlusion and normal breathing during taste perception. While subject-independent generalization remains limited (Section 5.4), these rules identify consistent within-sample neural signatures that can serve as hypotheses for future, larger-scale studies.

### 5.4. Between-Subjects Design Limitations

The LOSO results demand direct and transparent discussion. All methods, including the proposed framework and all baselines, achieve near-chance performance under LOSO cross-validation. This outcome is not a failure of the proposed method specifically; it is a structural consequence of the between-subjects design.

In this dataset, five participants are in the nose-opened condition and five are in the nose-closed condition. In a LOSO fold leaving out one closed-nose subject, the training set contains no closed-nose subjects whatsoever, making it structurally impossible to learn condition-specific features for that class. The five closed-nose subjects each receive 0.0% F1 uniformly across all methods, confirming that this is a data structure problem, not a model problem. Among the five opened-nose subjects, performance is highly variable (KK: 85.3%; HIO: 15.0%), reflecting the substantial inter-individual EEG variability that is well-documented in the literature [1,3].

The 89.50% stratified accuracy represents a genuine classification of EEG patterns within this participant pool, likely combining condition-related neural activity with individual EEG baseline differences. This is the standard evaluation paradigm in the EEG feature extraction literature, which is appropriate for assessing method capability in controlled settings. The permutation test (*p* > 0.05) confirms that the observed LOSO performance is consistent with chance, ruling out statistically significant cross-subject condition discrimination in this sample.

Future work should employ within-subjects designs, where the same participants complete both conditions, enabling a direct within-person comparison that eliminates between-group confounds. Alternatively, substantially larger between-subjects samples (*N* ≥ 30 per condition) with careful demographic matching may enable cross-subject generalization. The evolutionary-fuzzy framework remains a strong candidate for both scenarios, given its demonstrated feature stability and interpretability.

### 5.5. Interpretation of Noise Introduction and Future Directions

The 19.6% F1 degradation from 0% to 30% noise (Table 5) reflects the high information density of the 25 evolved features. Each feature contributes substantially to the classification, so corruption of any individual feature has greater impact than for full 612-feature models where noise averages out across redundant features. This is an inherent trade-off of aggressive dimensionality reduction. Potential mitigations include ensemble approaches that aggregate multiple compact feature sets, noise-augmented training during the ES fitness evaluation, or robust fuzzy membership designs that are less sensitive to input perturbations. These represent productive directions for future work. Future research could enhance this framework by employing a noise-aware evolutionary fitness evaluation, where noise is dynamically injected during the training phase to naturally evolve highly robust fuzzy membership representations.

## 6. Conclusions

We have presented an evolutionary-fuzzy feature extraction framework for EEG classification that prioritizes interpretability and compact representation. The method achieves 88.31% F1 with 25 features, showing a 95.9% dimensionality reduction. The method has a smaller overfitting gap than comparable 25-feature baselines, and produces fully interpretable fuzzy rules that are directly tied to neurophysiologically meaningful signal features. Applied to a chemosensory EEG experiment, the framework consistently identifies frontal and parietal beta-band and temporal dynamics features across 20 independent runs, demonstrating strong reproducibility.

We provide a comprehensive and transparent validation: stratified split comparison with fair feature-count-matched baseline, noise robustness analysis, and honest LOSO assessment acknowledging the generalization limits of the between-subjects design. This transparency is itself a methodological contribution, demonstrating what honest validation of small between-subjects EEG studies should include.

The evolutionary-fuzzy framework is applicable to any EEG classification task requiring interpretable, compact feature representations. Its combination of automated nonlinear feature discovery, fuzzy linguistic encoding, and evolution strategy optimization provides a principled, reproducible route from high-dimensional raw EEG to human-readable classification rules. It is important to note that due to the small, between-subjects sample size where subject identities are inherently confounded with class labels, the high accuracy reported reflects within-dataset discrimination, rather than true cross-subject generalization. The primary contribution of this work lies in the methodological demonstration of the evolutionary-fuzzy framework’s ability to extract interpretable and compact features. Given the sample constraints, the neurophysiological patterns identified by the interpretable fuzzy rules should be viewed strictly as hypothesis-generating observations, rather than definitive neuroscientific conclusions regarding chemosensory processing.

## Figures and Tables

**Table 1:** Top-performing hyperparameter configurations for the evolutionary-fuzzy framework.

Rank	*N_trees_*	*N_extracted_*	*N_selected_*	μ/λ	F1 (%)
1	300	25	25	40/120	89.41 ± 2.54
2	300	20	20	40/120	88.91 ± 1.71
3	300	25	20	30/90	88.32 ± 2.24
4	300	25	20	27/100	88.04 ± 1.89
5	300	25	20	40/60	87.81 ± 2.47

**Table 2:** Performance comparison between the proposed evolutionary-fuzzy framework and machine learning methods.

Method	Train F1 (%)	Test F1 (%)	Gap (%)	Polygon Area
SVM-Linear (612 feat.)	100.00 ± 0.00	97.87 ± 0.65	+2.13	0.9512 ± 0.0147
SVM-RBF (612 feat.)	99.85 ± 0.05	98.49 ± 0.52	+1.36	0.9654 ± 0.0117
RF-612 (612 feat.)	100.00 ± 0.00	98.91 ± 0.51	+1.09	0.9748 ± 0.0117
k-NN (612 feat.)	98.08 ± 0.28	96.17 ± 0.69	+1.90	0.9132 ± 0.0150
RF-CFS-25 (fair control)	100.00 ± 0.00	93.57 ± 1.14	+6.43	0.8587 ± 0.0236
Proposed (Evo-Fuzzy + RF)	90.50 ± 1.66	88.31 ± 2.43	+2.20	0.7759 ± 0.0491

**Table 3:** Top 10 most frequently selected raw features by the CFS algorithm.

Rank	Feature Index	Domain	Channel	Metric
1	9	Time Domain	Ch1	*ZCR*
2	11	Time Domain	Ch1	*AccRMS*
3	53	Time Domain	Ch5	*ZCR*
4	54	Time Domain	Ch5	*VelRMS*
5	132	Time Domain	Ch12	*AccRMS*
6	143	Time Domain	Ch13	*AccRMS*
7	154	Time Domain	Ch14	*AccRMS*
8	191	Frequency Domain	Ch1	*Beta*
9	194	Frequency Domain	Ch1	*SpecCentroid*
10	195	Frequency Domain	Ch1	*SpecSpread*

**Table 4:** Sample fuzzy rules from the best-performing run.

Rule	Antecedent (IF…)	Consequent (THEN…)
1	(Ch1-ZCR is LOW) AND (Ch1-SpecCentroid is MEDIUM)	Nose-Opened
2	(Ch5-AccRMS is HIGH) AND (Ch12-AccRMS is HIGH)	Nose-Closed
3	(Ch1-Beta is HIGH) AND (Ch5-ZCR is LOW)	Nose-Opened
4	(Ch13-AccRMS is HIGH) AND (Ch1-ZCR is MEDIUM)	Nose-Closed
5	(Ch1-SpecSpread is LOW) AND (Ch5-VelRMS is MEDIUM)	Nose-Opened

**Table 5:** Noise robustness: Test F1 (%) under gaussian noise.

Method	0% Noise	10% Noise	20% Noise	30% Noise	Δ
RF-612	98.91 ± 0.51	98.67 ± 0.56	98.31 ± 0.66	97.26 ± 0.80	−1.65
RF-CFS-25	93.57 ± 1.14	93.06 ± 1.37	92.19 ± 1.39	90.63 ± 1.56	−2.94
Proposed	88.31 ± 2.43	79.26 ± 3.50	73.33 ± 3.74	68.70 ± 4.36	−19.6

## Data Availability

The evolutionary-fuzzy codebase and custom feature extraction scripts of this study will be made available in a public repository upon publication.

## References

[1] Naser A., Aydemir Ö. (2024). Enhancing EEG signal classification with a novel random subset channel selection approach: Applications in taste, odor, and motor imagery analysis. IEEE Access.

[2] Ergün E., Aydemir Ö., Korkmaz O.E. (2025). Investigating the informative brain region in multiclass electroencephalography and near infrared spectroscopy based BCI system using band power based features. Comput. Methods Biomech. Biomed. Eng..

[3] Swiegers J.H., Chambers P.J., Pretorius I.S. (2005). Olfaction and taste: Human perception, physiology and genetics. Aust. J. Grape Wine Res..

[4] Lipton Z.C. (2018). The mythos of model interpretability. Queue.

[5] Rudin C. (2019). Stop explaining black box machine learning models for high stakes decisions and use interpretable models instead. Nat. Mach. Intell..

[6] Arrieta A.B., Díaz-Rodríguez N., Del Ser J., Bennetot A., Tabik S., Barbado A., Garcia S., Gil-Lopez S., Molina D., Benjamins R. (2020). Explainable Artificial Intelligence (XAI): Concepts, taxonomies, opportunities and challenges toward responsible AI. Inf. Fusion.

[7] Yarlagadda V.K. (2021). Harnessing biomedical signals: A modern fusion of hadoop infrastructure, AI, and fuzzy logic in healthcare. Malays. J. Med. Biol. Res..

[8] Mollo E., Boero F., Peñuelas J., Fontana A., Garson M.J., Roussis V., Cerrano C., Polese G., Cattaneo A.M., Mudianta I.W. (2022). Taste and smell: A unifying chemosensory theory. Q. Rev. Biol..

[9] Kato M., Okumura T., Touhara K., Okamoto M. (2025). Behavioral relevance of early neural coding of low-level odor features in humans. J. Neurosci..

[10] Prescott J. (2012). Chemosensory learning and flavour: Perception, preference and intake. Physiol. Behav..

[11] Chen Y.P., Ding Z., Yu Y., He P., Zhou Y., Liu Y., Feng X. (2023). Recent advances in investigating odor-taste interactions: Psychophysics, neuroscience, and microfluidic techniques. Trends Food Sci. Technol..

[12] Morozova M., Bikbavova A., Bulanov V., Lebedev M.A. (2023). An olfactory-based brain-computer interface: Electroencephalography changes during odor perception and discrimination. Front. Behav. Neurosci..

[13] Pereira D.R., Pereira H.R., Silva M.L., Pereira P., Ferreira H.A. (2025). Impact of five basic tastes perception on neurophysiological response: Results from brain activity. Food Qual. Prefer..

[14] Andersen C.A., Kring M.L., Andersen R.H., Larsen O.N., Kjær T.W., Kidmose U., Møller S., Kidmose P. (2019). EEG discrimination of perceptually similar tastes. J. Neurosci. Res..

[15] Wang Z., Chang X., Zhang C., Lan H., Huang M., Zhou B., Sun B. (2025). Beyond aromas: Exploring the development and potential applications of electroencephalography in olfactory research. Annu. Rev. Food Sci. Technol..

[16] Wu W., Xu C., Liang Q., Zheng X., Xiao Q., Zhong H., Chen N., Lan Y., Huang X., Xie Q. (2023). Olfactory response is a potential sign of consciousness: Electroencephalogram findings. Front. Neurosci..

[17] Maeda S., Yoshimura H. (2019). Enhancement of electroencephalogram activity in the theta-band range during unmatched olfactory-taste stimulation. J. Physiol. Sci..

[18] De S., Mukherjee P., Roy A.H. (2025). TasteNet: A novel deep learning approach for EEG-based basic taste perception recognition using CEEMDAN domain entropy features. J. Neurosci. Methods.

[19] Cybenko G. (1989). Approximation by superpositions of a sigmoidal function. Math. Control Signals Syst..

[20] Box G.E.P., Cox D.R. (1964). An analysis of transformations. J. R. Stat. Soc. Ser. B.

[21] Small D.M., Prescott J. (2005). Odor/taste integration and the perception of flavor. Exp. Brain Res..

[22] Simon S.A., de Araujo I.E., Gutierrez R., Nicolelis M.A. (2006). The neural mechanisms of gustation: A distributed processing code. Nat. Rev. Neurosci..

[23] Aydemir O. (2021). A new performance evaluation metric for classifiers: Polygon area metric. J. Classif..

